# Obsessive-Compulsive Disorder With Rheumatological and Inflammatory Diseases: A Systematic Review

**DOI:** 10.7759/cureus.14791

**Published:** 2021-05-01

**Authors:** Ahmed M Alsheikh, Maram M Alsheikh

**Affiliations:** 1 Medicine, College of Medicine, Almaarefa University, Riyadh, SAU; 2 Epidemiology and Public Health, Faculty of Medicine, Suez Canal University, Ismalia, EGY

**Keywords:** rheumatology, obsessive-compulsive disorders, inflammation, association, biomarkers, systematic review

## Abstract

Obsessive-compulsive disorder (OCD) is a common mental illness that can significantly impair the patients' quality of life. Recent studies have shown that patients with this condition usually suffer from inflammatory or rheumatological comorbidities. However, the association between OCD's etiology and inflammation is still controversial. This review aims to explore the correlation between OCD and rheumatological as well as inflammatory disorders based on studies conducted in the last decade. A total of eight articles that were deemed eligible were included in the final assessment, involving 31,204 OCD patients from various countries. The most significant inflammatory biomarkers examined were tumor necrosis factor-alpha (TNF-α), interleukins, neutrophil-to-lymphocyte ratio (NLR), and cytokines. We concluded that the pathophysiology and etiology of OCD are strongly correlated with inflammatory biomarkers. This finding warrants future studies on the efficacy of anti-inflammatory agents to treat OCD, particularly in the early stages of the disease.

## Introduction and background

Obsessive-compulsive disorder (OCD) is defined as a severe mental disease characterized by recurrent compulsions and obsessions that can lead to significant stresses and functional abnormalities [1]. It is estimated that 2% of the population globally are affected by OCD [2]. There is no significant difference between genders regarding the prevalence of the condition; however, it commonly affects those under 20 years of age, particularly males [3]. The etiology of OCD is still unclear, yet it is proposed to be a complicated process, which involves an interaction between multiple factors. These include genetic, inflammatory, and environmental facets [4]. Some studies have recently speculated that the pathogenesis of OCD could be linked to autoimmune and rheumatological disruptions [5]. Additionally, there is increasing evidence about the rising number of OCD patients reporting rheumatological comorbidities [6]. In the pediatric population, OCD has been linked to some antibodies for streptococcal infections, which can cross-react with other epitopes found on the neurons of the basal ganglia. Most commonly, this cross-reaction results in pediatric acute-onset neuropsychiatric syndrome (PANS) as well as pediatric autoimmune neuropsychiatric disorders associated with streptococcal infections (PANDAS) [7,8]. 

Multiple inflammatory and rheumatological markers have been studied among patients with OCD [9]. Recent studies have demonstrated a potential correlation between circulating cytokines and OCD diagnosis [10]. Gray and Bloch studied proinflammatory cytokines markers in OCD patients by performing a systematic review [11]. They reported that tumor necrosis factor-alpha (TNF-α) marker was found elevated in OCD patients [11]. Additionally, it has been shown that patients who do not receive treatment for OCD usually have higher levels of inflammatory biomarkers compared to their peers who are on treatment [12]. The inflammatory and rheumatological abnormalities were examined in both children and adults with almost similar outcomes [13]. Also, abnormalities in inflammatory biomarkers' production pathways have been detected in OCD patients, particularly cytokines, monocytes, and interleukins [13]. Despite the growing interest in the last five decades in understanding the etiology and pathogenesis of OCD, there is a scarcity of data to confirm the clinical association between the pathogenesis of OCD along with the severity of its symptoms and the levels of inflammatory as well as rheumatological biomarkers, which deserve further exploration. Accordingly, the present review aims to thoroughly examine the medical literature published during the last decade to explore the correlation of OCD with inflammatory and rheumatological diseases.

## Review

The present systematic review was performed as per the Preferred Reporting Items for Systematic Reviews and Meta-Analyses (PRISMA) checklist recommendations [14]. We conducted a search of the following electronic databases to find eligible trials from January 2010 till November 2020: Medline, PsycINFO, Embase, and PubMed.

Search strategy

We used the following Medical Subject Headings (MeSH) terms to elicit relevant results: "obsessive-compulsive disorders" OR "OCD" AND "Inflammatory" AND "rheumatology” AND “Association” OR “correlation” OR “link”. All the titles and abstracts that resulted from this initial search were revised thoroughly to avoid missing any pertinent studies. The outcomes were then refined to include only original research articles investigating the association between OCD and inflammatory or rheumatological diseases and markers among adults or pediatric patients. Selected trials mentioned the population under investigation and the type of examined biomarkers. Additionally, all studies from various countries were included. Only those studies published in English were selected, which would be further evaluated in the second step.

Eligibility criteria

**Figure 1:**
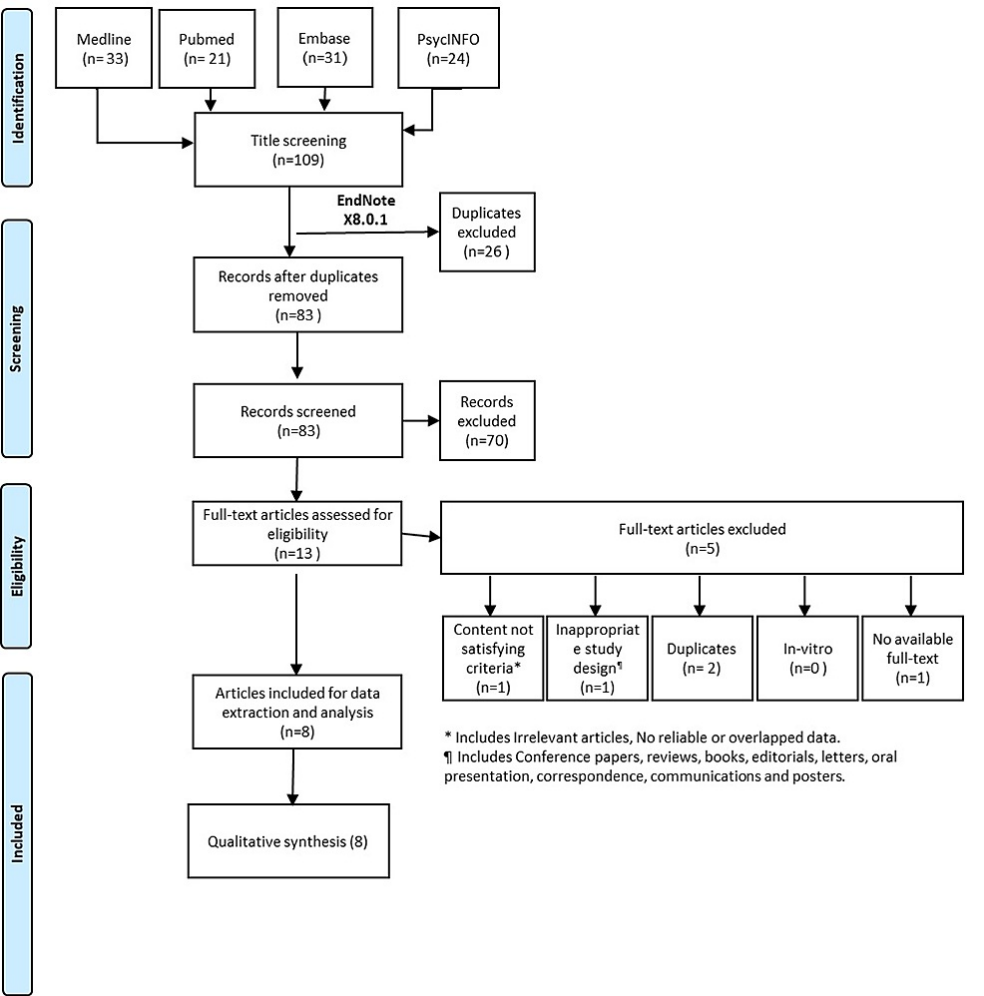
PRISMA flowchart summarizing the search process in this study PRISMA: Preferred Reporting Items for Systematic Reviews and Meta-Analyses

Abstracts were evaluated manually to select the relevant ones for consideration. The inclusion criteria were as follows: articles that contained information on the study population and examined inflammatory or rheumatological markers. Moreover, the reference section of the selected studies was examined to find any additional relevant studies. Finally, the pre-defined data sets were gathered from the final record of eligible articles and summarized. Articles were excluded if they were review studies, overlapped with other articles, had incomplete data, or in cases of unavailability of full-text articles or inappropriate study design. The flowchart illustrating the search strategy is presented in Figure 1.

Data review

Two reviewers drafted a specially designed Excel sheet, which was used for data extraction. Afterward, selected data from eligible studies were revised through the Excel sheet. Any articles published by one research group investigating similar variables were reviewed for any possible duplication. Any discrepancies were resolved through a discussion with a senior member.

Results

After searching the abstracts and applying the eligibility criteria on identified potential abstracts, eight articles published between January 2010 and November 2020 were included in the present systematic review, involving 31,204 OCD patients from different countries. Out of the eight studies [15-22], five were randomized controlled studies [16,17,20-22], while two studies were retrospective [15,19], and one study was a total-population multigenerational family cohort study [18]. Furthermore, the pediatric population was examined in three papers [17,19,20], while the adult population was examined in four articles [15,16,21,22], and both adults and pediatrics were examined in one multi-generation study [18].

It has been shown that the levels of inflammatory and rheumatic biomarkers are significantly elevated in both pediatric and adult patients with OCD. The most significant biomarkers examined were TNF-α, interleukins, neutrophil-to-lymphocyte ratio (NLR), and cytokines. These biomarkers may also be related to a genetic predisposition to the condition, as described in detail in Table 1.

**Table 1:** Summary of the included studies BDNF: brain-derived neurotrophic factor; CVI: choroidal vascularity index; FED: fluoxetine equivalent-dose; GM-CSF: granulocyte-macrophage colony-stimulating factor; NRL: neutrophil-to-lymphocyte ratio; OCD: obsessive-compulsive disorder; PANDAS: pediatric autoimmune neuropsychiatric disorders associated with streptococcal infections; PANS: pediatric acute-onset neuropsychiatric syndrome; sTNFR1: soluble tumor necrosis factor receptor-1; sTNFR2: soluble tumor necrosis factor receptor-2; TGFβ: transforming growth factor beta; PLR: platelet-to-lymphocyte ratio; TNF-α: tumor necrosis factor-alpha; NSAIDs: non-steroidal anti-inflammatory drugs

Author(s)	Year	Study design	Sample size	Population	Objective	Results
Herdi et al. [15]	2020	Retrospective Study	135	Adults	To examine the correlation between OCD and subclinical inflammatory markers, NLR and PLR	NLR and PLR were significantly higher in OCD. Contrary to the correlation of FED with NLR, PLR did not correlate with FED. PLR is a robust biomarker to medication effect contrary to NLR. Both NLR and PLR significantly predicted OCD
Sekeryapan et al. [16]	2020	Randomized controlled study	64	Adults	To investigate the association of CVI with the NLR as an indicator of inflammation in OCD	The subfoveal choroidal thickness, peripapillary CT, and CVI values were significantly higher in the OCD than in the control group (p˂0.05). The NLR values were significantly higher in the OCD group (p=0.007). A significant positive correlation was noted between CVI and NLR (p=0.039). Systemic inflammation could have a role in the pathogenesis of OCD
Sivri et al. [17]	2018	Randomized controlled study	84	Pediatrics	To investigate interleukin-12, interleukin-17, TGFβ, TNF-α, sTNFR1, sTNFR2, interleukin-1β, CCL3, CCL24, CXCL8, and BDNF with OCD in medication-free children	There was a significant difference in OCD patients in cytokine, chemokine serum levels, an effect that was independent of severities of OCD (p<0.001). The serum TNF-α levels were significantly higher in the OCD group (p<0.001). On the other hand, serum interleukin-12 levels were significantly lower in the OCD group than in the control group (p=0.014). These findings suggest that TNF-α and interleukin-12 may play a role in the pathophysiology of OCD in children
Mataix-Cols et al. [18]	2018	Total-population multigenerational family cohort study	30,082	Pediatrics and adults	To evaluate the association between OCD and rheumatoid diseases	OCD patients had increased comorbidity with any rheumatic disease (43%). The risk of any rheumatoid diseases was consistently higher among first-degree relatives than among second-and third-degree relatives of OCD patients. The risk of rheumatoid diseases was very similar in mothers, fathers, and siblings of OCD patients. There was a familial link between rheumatoid diseases in general (that is, not limited to *Streptococcus*-related conditions) and OCD
Spartz et al. [19]	2017	Retrospective study	218	Pediatrics	To describe OCD in patients diagnosed with PANS and PANDAS after introducing or removing NSAIDs	31% had an improvement in OCD symptoms; 35% had escalation in OCD; 39% experienced side effects, mainly mild gastrointestinal symptoms, which self-resolved after removing NSAIDs, reducing the dose, or changing NSAIDs. Improvement in OCD symptoms was evident in one-third of NSAID treatment trials
Rodríguez et al. [20]	2017	Randomized controlled study	149	Pediatrics	To study inflammatory markers in monocytes with OCD	OCD patients had significantly higher percentages of total monocytes and CD16+ monocytes. Monocytes from OCD patients released higher amounts of GM-CSF, interleukin-1β, interleukin-6, interleukin-8, and TNF-α than healthy controls after exposure to lipopolysaccharides. However, there were no significant differences in basal cytokine production or the sensitivity of monocytes to dexamethasone treatment between both groups. Based on monocyte subset distribution and cytokine production after lipopolysaccharides stimulation, patients receiving psychoactive medications seemed to have an intermediate inflammatory profile, lower than non-medicated OCD individuals and higher than healthy controls. There was an involvement of an enhanced proinflammatory innate immune response in the etiopathogenesis of early-onset OCD
Rao et al. [21]	2015	Randomized controlled study	40	Adults	To explore plasma cytokine abnormalities in patients with OCD	OCD patients had significantly greater plasma levels of interleukin-2, interleukin-4, interleukin-6, interleukin-10, and TNF-α levels than controls but not IFN-γ. The presence of these abnormalities in drug-naïve patients suggests the possible role of cytokines in the pathogenesis of OCD. Study findings have potential clinical utility in the development of novel therapeutic options targeting cytokine aberrations in OCD
Cappi et al. [22]	2012	Randomized controlled study	432	Adults	To explore the association between functional polymorphisms in the TNF-α gene and OCD	The A allele of the TNF-α rs361525 polymorphism was significantly associated with OCD subjects (p=0.007). The presence of genetic markers, such as inflammatory cytokines genes linked to OCD, may represent additional evidence supporting the role of the immune system in its pathogenesis

Discussion

Primary OCD is considered a clinically heterogeneous disorder with unknown etiologies typically starting in childhood and adolescence [23]. OCD is one of the most common mental illnesses that has been proposed to be linked to other systemic diseases. Additionally, many factors have been linked to OCD, such as environmental, hereditary, and genetic facets. Some studies have found a correlation between OCD and inflammatory as well as rheumatic diseases. However, this correlation is still not fully established. Hence, understanding the inflammatory pathophysiology of OCD can guide the development of novel treatment strategies for the disease. The present review examines the correlation between OCD and rheumatological as well as inflammatory disorders. The review has demonstrated a significant increase in inflammatory and rheumatic biomarkers in patients with OCD, especially before starting OCD treatment (treatment-naïve patients). The most significant inflammatory biomarkers were TNF-α, interleukins, NLR, and cytokines.

In adults, the correlation between OCD and inflammation has been examined in four studies. Most recently, Herdi et al. [15] illustrated significantly higher levels of inflammatory biomarkers in patients with OCD. They also showed that platelet-to lymphocyte ratio would be a more accurate biomarker to indicate the effect of OCD medications [15]. However, it is worth mentioning that theirs was a retrospective study, which could limit its findings. Sekeryapan et al. [16] conducted another randomized controlled study that examined the correlation of two inflammatory indicators [NLR and choroidal vascularity index (CVI)] with OCD. They reported a significant increase in the level of inflammatory indicators in patients with OCD (p<0.05). These findings support the hypothesis that points to an inflammatory etiology of OCD. Other inflammatory mediators were investigated by Rao et al. [21] and Cappi et al. [22]. Rao et al. examined interleukin levels and TNF-α levels in patients with OCD who had not started their treatment for the condition. The study revealed significantly higher levels of these mediators in OCD patients, which could be explained by the involvement of cytokines in the pathophysiology of OCD. Cappi et al. also demonstrated a particular TNF polymorphism (an allele of TNF-α rs361525) that is significantly dominant in OCD patients compared to control.

The findings of Cappi et al. [22] highlighted the correlation between inflammatory genetic markers and OCD pathogenesis. The genetic correlation between inflammation and OCD was also supported by Mataix-Cols et al. [18] who examined multiple generations of pediatrics and adults in a study that recruited the highest number of OCD patients to date (30,082 patients). They demonstrated that almost half of OCD patients (43%) had rheumatic comorbidity, with a significantly higher incidence of rheumatic comorbidity among first-degree relatives of patients with OCD. Sivri et al. [17] demonstrated a significantly higher level of cytokine, chemokine, and TNF-α in children with OCD. However, in contrast to Rao et al.'s [21] findings among adults, Sivri et al. [17] showed significantly lower interleukin levels in children with OCD. These outcomes have led to a better understanding of the pathophysiology of OCD in pediatrics.

The findings of Sivri et al. [17] among pediatrics were contradicted by Rodríguez et al. [20], who examined the inflammatory markers among OCD children. Rodríguez et al. showed significantly elevated levels of total monocytes, including interleukins and TNF-α. Additionally, patients who received psychoactive medications had a lower inflammatory profile. Accordingly, early-onset OCD or naïve OCD patients might have significantly higher inflammatory biomarkers than those who received treatment. Supporting the correlation between OCD and inflammation, Spartz et al. [19] showed that the addition of non-steroidal anti-inflammatory drugs (NSAIDs) to the conventional psychoactive treatment of pediatrics with OCD could improve their symptoms. However, NSAIDs' side effects should be considered on a long-term basis in this patient population.

The present review has some limitations. Although the included studies examined the correlation between OCD etiology and inflammatory or rheumatic biomarkers, only one study examined the impact of NSAIDs and other anti-inflammatory medications as part of the treatment plan for OCD patients. This requires further investigations, preferably through studies with a robust design. Furthermore, studies with pre-existing psychiatric conditions in subjects were not included as it was difficult to assess. Therefore, studies that explore pre-existing conditions might reach conclusions that differ from ours.

## Conclusions

Based on our findings, there is a strong correlation between OCD and different inflammatory as well as rheumatological biomarkers. Such correlations have been demonstrated in both adult and pediatric populations. Future studies should examine the safety and efficacy of anti-inflammatory medications, especially NSAIDs, for the management and control of OCD symptoms in both adults and children, particularly in the early stages of the disease.
